# Designing Consumer Health Technologies for the Treatment of Patients With Depression: A Health Practitioner's Perspective

**DOI:** 10.2196/ijmr.2368

**Published:** 2014-01-10

**Authors:** Ginger White, Kelly Caine, Kay Connelly, Rebecca Selove, Tom Doub

**Affiliations:** ^1^School of Informatics and ComputingIndiana UniversityBloomington, INUnited States; ^2^School of ComputingClemson UniversityClemson, SCUnited States; ^3^Centerstone Research InstituteBloomington, INUnited States

**Keywords:** depression, health care providers, technology, user-computer interface

## Abstract

**Background:**

The consumer health technologies used by patients on a daily basis can be effectively leveraged to assist them in the treatment of depression. However, because treatment for depression is a collaborative endeavor, it is important to understand health practitioners’ perspectives on the benefits, drawbacks, and design of such technologies.

**Objective:**

The objective of this research was to understand how patients and health practitioners can effectively and successfully influence the design of consumer health treatment technologies for treating patients with depression.

**Methods:**

A group of 10 health practitioners participated in individual semistructured contextual interviews at their offices. Health practitioners rated an a priori identified list of depression indicators using a 7-point Likert scale and generated a list of depression indicators. Finally, health practitioners were asked to rate the perceived usefulness of an a priori identified list of depression treatment technologies using a 7-point Likert scale.

**Results:**

Of the 10 health practitioners interviewed, 5 (50%) were mental health practitioners, 3 (30%) nurses, and 2 (20%) general practitioners. A total of 29 unique depression indicators were generated by the health practitioners. These indicators were grouped into 5 high-level categories that were identified by the research team and 2 clinical experts: (1) daily and social functioning, (2) medication, (3) nutrition and physical activity, (4) demographics and environment, and (5) suicidal thoughts. These indicators represent opportunities for designing technologies to support health practitioners who treat patients with depression. The interviews revealed nuances of the different health practitioners’ clinical practices and also barriers to using technology to guide the treatment of depression. These barriers included (1) technology that did not fit within the current practice or work infrastructure, (2) technology that would not benefit the current treatment process, (3) patients forgetting to use the technology, and (4) patients not being able to afford the technology.

**Conclusions:**

In order to be successful in the treatment of depression, consumer health treatment technologies must address health practitioners’ technology concerns early on in the design phase, account for the various types of health practitioners, treatment methods, and clinical practices, and also strive to seamlessly integrate traditional and nontraditional depression indicators within various health practitioners’ clinical practices.

## Introduction

### Overview

Depression is a mental illness that affects millions of Americans every year [1]. Both adolescents and adults, spanning almost all ethnic and economic backgrounds, fall into depression. Depression is defined as a mood disturbance characterized by a depressed mood for a duration of 2 weeks or more or loss of interest or pleasure, and at least 4 other symptoms such as a change in functioning such as sleeping and eating habits, energy levels, and self-image [2]. Although the symptoms greatly vary between individuals and can change throughout the duration of depression, depressed individuals typically experience symptoms such as fatigue, feelings of hopelessness and helplessness, change in sleeping patterns, inability to concentrate or make decisions, change in appetite and energy levels, withdrawal, and thoughts of suicide [2].

Individuals affected by depression who seek treatment typically are prescribed medication or undergo in-person psychotherapy sessions with a professional [3]. However, a recent trend is observed among consumers who use online or mobile mental health treatment technologies with the intention of eliminating social stigma [4] and preserving anonymity [5]. Because the treatment of depression, in both online and office settings, is a collaborative effort between the patient and the physician, it becomes important to understand the needs of both. It is also essential to understand the perspective of several types of physicians since patients often receive care from different types of practitioners depending on their needs, established relationships [6], and comfort levels.

Currently, user adoption of consumer health treatment technologies is promising and more researchers are seeking to improve upon existing technologies. Although some researchers have presented guidelines to aid in the development of health technologies [7-9], only a few have incorporated the perspectives of various types of health practitioners. The aim of the present research is to understand how insights from health practitioners who treat depression may possibly inform future designs of consumer health technologies that may be used for treating depression. This study also helps to analyze how current successful patient-empowering treatment plans that are developed by both patient and the physician can translate into future consumer health technologies, as well as to determine how these technologies can be better designed to fit both the needs of the patient and the physician.

In 2009, approximately 6.1 million adults (aged 18 years or older) in the United States reported an unmet need or did not receive care or services for their mental health needs [1]. Unfortunately, many depressed individuals are unaware of the various treatment services available [3]. In the following sections, an overview of these treatment services is provided. The first section focuses on the different types of health practitioners who provide treatment for depression. This section discusses different types of mental health providers and the frequency and characteristics of services offered by them. The second section presents an overview of the technologies that are emerging as potential treatment options for depression.

### Types of Mental Health Providers

Most individuals who require treatment for their mental health problems seek help from their family physician or go to nearby emergency facilities [6]. As a result, general practitioners increasingly feel a need to train themselves to assess not only the physical symptoms of patients but also the psychological aspects [10]. Traditionally, nurses and general practitioners are trained to assess the physical aspects of a patient and therefore often overlook any psychological symptoms that may be present [11]. Office visits to medical practitioners tend to be shorter than that to mental health practitioners, and importantly, the diagnosis lacks psychological content [12]. Most nurses and general practitioners refer patients with depression to mental health practitioners. At the point of referral, patients’ medical and physical symptoms are assessed and reviewed, so the mental health practitioner can review the physical symptoms and concentrate on the psychological aspect of depression. Mental health practitioners spend their initial therapy sessions by engaging in get-to-know-you sessions with the patients [13]. These sessions are often longer than the usual standard office visits with medical practitioners, involve questioning related to everyday responsibilities and challenges, and focus more on understanding the events and causes that led to the patient’s depression [14]. After these in-depth sessions, mental health providers can build rapport with patients and acquire information that patients are usually not comfortable sharing. The longer sessions with mental health practitioners are ideal for facilitating recovery from depression [15].

### Emerging Technologies for the Treatment of Depression

In the recent years, many technologies have been developed to help track and understand the physical and psychological aspects of mental illness. Some of them help assess severity [16] and sometimes aid in the treatment of depression [17-21]. Some technologies monitor specific symptoms of depression such as mood and behavior changes [22-24], quality and quantity of sleep [25-27], eating habits [28], and physical activity [29]. Many of these technologies, especially online treatment applications, enjoy positive patient adoption and provide satisfactory solutions [30,31]. Many patients opt to use such technologies so that they can remain anonymous, thus avoiding the stigma and embarrassment that they have to endure while seeking treatment from a traditional behavioral health center [5]. These technologies also allow individuals to seek treatment from the comfort of their home [32] and avoid what could be costly in-person treatment and services [33].

Although many of these health technologies have been adopted by patients independently, a few mental health technologies have been successfully influenced by clinicians. These technologies are not employed with the assistance or presence of a mental health or medical practitioner, or not integrated within the treatment plan. However, if the technologies are to be included as part of the overall therapy plan, the needs and perspectives of the health practitioners who treat depression must be understood.

### Aims of the Study

Information gathering and context-sensitive technologies hold a great potential for making depression treatment more effective. However, prior research has focused almost exclusively on technology development, and to a lesser extent on patient’s perspectives on technology adoption outside of the clinical setting. Only a few studies focus on understanding the clinicians’ perspectives with respect to treating depression [34-36], and even fewer studies examine the differences in needs and practices between health practitioner types [37,38]. This paper presents results from a study examining how various clinical styles of treating depression can potentially inform the design of useful patient-empowering consumer health treatment technologies.

## Methods

### General Method

The qualitative contextual interview method [39] was chosen to examine the current method of treatment of individuals suffering from behavioral health issues and to gain feedback about potential technologies that may be integrated to a treatment regime. The main reason for choosing the contextual interview method is because health practitioners’ perceptions, in particular differences in perceptions between health practitioner types, have not been systematically studied. In this study, we used qualitative inquiry to gather the local vocabulary and practices of the 3 health practitioner types: 3 nurses, 2 general practitioners, and 5 mental health practitioners. In addition, as we were interested in understanding the differences between health practitioner types, it was important to limit this investigation to the study of participants from within a single organization and region to the standardize organizational culture and treatment population. Those nuances would be hard to tease out and isolate using a study method such as a questionnaire that is targeted at a larger population, but are particularly sensitive to qualitative inquiry.

### Participants

A group of 10 health practitioners, including 3 (30%) nurses, 2 (20%) general practitioners, and 5 (50%) mental health practitioners volunteered to be part of this study. Hereafter, nurses and general practitioners will be referred to as medical practitioners. Mental health practitioners are defined as professionals who specialize in treating behavioral disorders. The age of health practitioners participating in the study varied between 29 and 61 years (mean 42.50, SD 10.98), and they had been practicing on an average for 10 years (mean 9.65, SD 7.08). Across all clinician types, the study participants reported that more than half (52.5%) of their patients suffered from depression. Clinicians reported using computers, the Internet, various rating scales, feedback systems, and intake assessments during the course of their work. Technologies used outside of the work included computers, the Internet, and smartphones. Prior to clinician recruitment for the study, institutional review board (IRB) approval was obtained from the Centerstone Research Institute IRB and was also reciprocally given by the Indiana University IRB.

### Procedure

Individual semistructured contextual interviews with each of the 10 study participants were conducted at their offices. Each interview lasted approximately 30 minutes. The interview focused on understanding the clinician’s perception of indicators related to patients’ depression status, and feedback was elicited on types of technology that could possibly be helpful in treating depression. The goal of the interviews was to understand clinicians’ actual practices rather than what they were “supposed” to do as indicated by training or organizational protocol. The authors defined indicators as the type of patient information that can be used to help treat depression. The first section of the interview focused on understanding current clinician practices in treating patients with depression. The second section of the interview focused on identifying the type of patient information clinicians would obtain from patients if it were possible to do so. Finally, clinicians were presented with technology concepts and asked to rate them in terms of perceived usefulness. Participants did not receive monetary remuneration for their participation in the study but were thanked for their participation.

### Daily Indicators

The first section of the interview focused on understanding current clinician practices for treating depression. This section consisted of 6 open-ended questions that were posed to health practitioners with a view of understanding the most relevant symptoms reported by patients and the type of patient information currently used in treating depression. Health practitioners were asked to rate their responses based on overall effectiveness in treating depression using a 7-point Likert-type scale. Following this rating, health practitioners were provided with a predetermined list of indicators by the research team. The list of indicators was compiled in collaboration with a team of clinical researchers and included suicidal ideation, sleep quality and quantity, social interactions, ability to focus, physical activity, service reimbursement method, and astrological sign. Participants were asked to rate the indicators based on overall effectiveness in treating depression. Responses were again rated using a 7-point Likert-type scale. The authors believed that collecting information on the bases of indicators would lead to insights, which in turn could be used to develop useful consumer health technologies.

### Technology Concepts

The goal of the second section of the interview was to gather health practitioners’ perceptions of the types of patient information health practitioners would be able to gain if a technology existed to enable them to collect and access this information. Participants were presented with 5 technology concepts and were asked to rate them in terms of perceived usefulness using a 7-point Likert scale. The technology concepts were gathered in collaboration with a team of clinical researchers and focused on covering a range of depression symptoms and feasible technologies. The technology concepts consisted of collecting information about patients’ home cleanliness, physical activity, social interactions, nutrition, and life space (movement in and outside of the home) (Table 1).

**Table 1:** Overview of technology concepts.

Technology concept	Motivation for depression	Implementation
Physical activity: Allows patients to track their physical activity levels and provide summary information to clinicians. 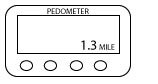	Individuals who are depressed typically show decreased physical activity [2]. Both clinicians and patients can be benefitted by understanding how the depression is affecting the physical activity level. Such information can be used in devising a treatment plan.	Several free and low-cost physical activity tracking applications are currently available on the Web and mobile devices. Pedometers are also popular low-cost devices used by individuals of all ages. Using already popular consumer-based health devices decreases the chance of having a stigma associated with using the devices.
Nutrition: Allows patients to track their nutrition intake and eating habits and provides summary information to clinicians. 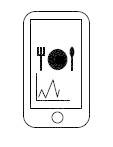	Individuals with depression are known to have a decrease in appetite [2] and run the risk of becoming malnourished. If information regarding one’s nutrition level was observed early and often, clinicians could design treatment plans that can prevent such incidents.	Currently, several online and mobile consumer health applications that focus on capturing one’s nutrition levels are present. Previous research has also shown successful implementation and adoption of consumer health devices that are designed to track nutrition levels [40].
LifeSpace: Records patients’ movement in and outside of their home and would provide clinicians with summary data about the locations where participants spend time. 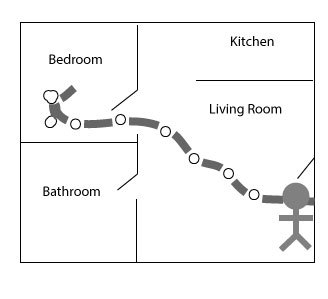	Individuals with depression often have a change in their mood and behavior [2], a decrease in mobility, and a change in their typical routine in and around the home. It has been shown that one’s ability to be mobile correlates directly with one’s overall health and mental status [41]. Being able to track these changes allows patients to observe how the depression is affecting their daily life and afford them the opportunity to make a change in their current coping mechanisms.	To implement a consumer health technology that can track one’s behavior and movement inside the home, video cameras or motion sensors can be installed around the home. To track movement outside of the home, one of the many several mobile global positioning system applications can be used.
Social Interactions: Measures the number of social interactions a patient has, the number of people with whom he or she interacts, and the amount of time spent with each individual. 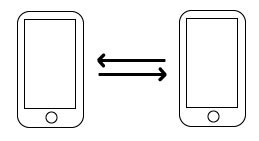	Depressed individuals often display some type of social withdrawal and even long periods of social isolation [2]. Having technology that can track one’s social activity level can potentially detect negative trends in one’s social activity level early on. Information such as this can potentially help individuals with depression seek intervention in a timely manner.	Technology such as Bluetooth and audio recognition could be utilized in a consumer health technology. Bluetooth technology detects Bluetooth-enabled devices in close proximity of the device of the patient with depression. This gives some information of the number of people who are possibly around the individual with depression. Additionally, one could utilize a microphone to pick up any audio frequency to determine if the individual with depression is engaged in any social activities or conversations.
Cleanliness (as an indicator of personal and household daily care): Allows patients to monitor their personal and household upkeep to understand a positive or negative change within their typical home routine. 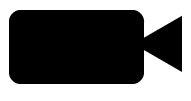	Individuals with depression often display a change in their typical routine and behavior [2]. This can lead to a decrease in one’s ability to maintain a positive sense of self and complete personal responsibilities in and around the home. If patients are able to track their ability to maintain their typical home routine, they can better understand how depression is affecting their home life and get any help they may need early on.	To implement a consumer health technology that can track an individual’s ability to complete personal responsibilities in and around the home, one can potentially utilize a video-monitoring device. For example, the device could capture samples of the patient’s home and then automatically analyze these samples to detect changes in the environment.

### Data Collection and Preparation

All interviews were audio recorded that took place at health practitioner offices. A member of the research team also took notes during each interview. Interviews were transcribed verbatim and then transcriptions were analyzed.

## Results

### Overview

The following section first presents quantitative results related to the indicators that health practitioners generated independently followed by the results of the health practitioner ratings to a set of a priori identified treatment indicators. Next, the results of health practitioner ratings of a priori identified technology concepts are presented. In the discussion section, the design implications related to each of these results are discussed.

### Health Practitioner-Generated Indicators

To get a complete understanding that is not limited by current clinical practice and guidelines, health practitioners were asked to generate a list of indicators that would be useful for treating depression. From this exercise, a total of 29 unique indicators were identified. These indicators were grouped into 7 high-level categories identified by the first 3 authors. The groupings were arrived at by consensus. Labels such as daily and social functioning, demographics and environment, medication, nutrition and health, and suicidal tendency were assigned to each category. Subsequently, 2 clinical experts from the research team were asked to label 29 unique indicators with one of the 7 high-level categories, or “other” with an associated description for what the “other” should be. This labeling activity reduced the number of categories to 5, in which daily status and social functioning were grouped into one category and demographics and environment into another. Based on the clinical experts’ labeling, 3 of the indicators were also moved from one category to another. The final 5 categories are shown in Table 2. For the purposes of the following analysis, nurses and general practitioners were combined into a medical practitioner group, resulting in 5 practitioners each in both the medical practitioner group and the mental health practitioner group.

Table 2 shows the indicators that the health practitioners believed would be helpful in guiding depression treatment. All of the health practitioners were interested in knowing more about patients’ daily and social functioning. Within the daily and social functioning category, medical practitioners generated items that were associated with the patient’s family and friends’ perspective on helping to treat depression, whereas the mental health practitioners generated items relating to patients’ support systems and social interactions. A larger percentage of mental health practitioners were particularly interested in medication used by the patients and their demographics and environment than were medical practitioners. Mental health professionals were also the only health practitioner type interested in patients’ suicidal risk. Medical and mental health practitioners were similarly interested in patients’ medication use, but more medical practitioners in the study group were interested in patients’ nutrition and physical activity levels than the mental health practitioners. Especially within the nutrition and physical activity category, medical practitioners were interested in the patients’ eating habits, whereas mental health practitioners were interested in the typical daily movement of the patients. Overall, health practitioners stated that patients’ changes in behavior would be less helpful in guiding treatment for depression.

**Table 2:** Clinician-generated indicators.

Indicator	Total mental health practitioner (N=5) n (%)	Total medical practitioner (N=5) n (%)	Total clinicians (N=10) n (%)
Daily and social functioning	5 (100)	5 (100)	10 (100)
Medication	2 (40)	2 (40)	4 (40)
Nutrition and physical activity	2 (40)	3 (60)	5 (50)
Demographics and environment	2 (40)	2 (40)	4 (40)
Suicidal tendency	3 (60)	0 (0)	3 (30)

### A Priori Identified Treatment Indicators

**Figure 1:**
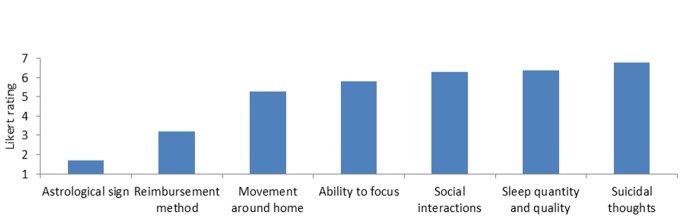
Perceived usefulness of indicators in guiding treatment.

To ensure cross-health practitioner type comparisons, depression indicators were identified by examining the literature associated with depression treatment and by collaborating with clinical experts. The indicators thus identified were chosen to represent a range of potential usefulness (eg, sleep was thought to be highly relevant, whereas astrological sign was thought to be irrelevant). To understand the level of usefulness of indicators, the Likert ratings that the clinicians provided for each of the 7 indicators were examined. Overall, clinicians believed that information concerning patient’s quantity and quality of sleep (mean 6.50, SD 0.71) and self-report of suicidal ideation (mean 6.80, SD 0.42) would be helpful in treating depression. Clinicians were less interested in using reimbursement method or astrological sign as essential information to treat depression (Figure 1).

### Technology Concepts

Table 3 shows health practitioners’ perceptions of the types of patient information that they could obtain if a technology existed to enable them to collect and access such information. In this section, general practitioners and nurses are not combined. Overall, tracking patients’ physical activity and social interactions were rated the highest by the health practitioners (numerically). In particular, general practitioners were very, interested (mean 7.00 and 6.50 and SD 0.00 and 0.71, respectively) in having a technology that allowed them to understand their depressed patients’ physical activities and social interactions. Health practitioners were interested in using a technology to track the nutrition status and cleanliness of the patients. General practitioners rated these technologies higher overall than either the mental health providers or the nurses. LifeSpace was rated as having the lowest utility overall, but had the greatest variability among ratings. Notably, nurses disagreed that the LifeSpace technology concept would be helpful to them in their practice (as this was the only technology that was rated unfavorable by any group); however, general practitioners strongly agreed that the LifeSpace technology concept would be helpful in their practice. The next section discusses these two sets of findings in the context of our qualitative results and uses them to suggest design opportunities.

**Table 3:** Mean rating of technology concepts (1=strongly disagree, 7=strongly agree).

Technology concept	Mental health practitioners (n=5) Mean (SD)	General practitioners (n=2) Mean (SD)	Nurses (n=3) Mean (SD)		Overall mean (N=10) Mean (SD)
Physical activities	5.40 (2.51)	7.00 (0.00)	5.30 (0.58)	4.80 (1.62)	
Social interactions	5.40 (2.61)	6.50 (0.71)	5.00 (0.00)	4.50 (2.51)	
Nutrition	5.00 (2.35)	6.00 (1.41)	4.00 (2.65)	5.70 (1.83)	
Cleanliness	4.40 (1.95)	5.50 (0.71)	5.00 (1.73)	4.90 (2.18)	
LifeSpace	4.60 (2.88)	6.50 (0.71)	3.00 (2.00)	5.50 (1.84)	

### Design Opportunities for Health Practitioners to Treat Patients With Depression

#### Overview

Interview discussions and health practitioner ratings of the list of a priori identified technology concepts clearly indicated that the health practitioners were favorable to using technology to help treat depression (Table 3). Health practitioners also expressed an interest in having technology that could assist in patient data collection.

So that’s why I encourage them to write things down, because they don’t have the information to bring to me, and if they could understand how to use technology and gather the information, then I think that would be great.

General practitioner 8

These findings suggest that there is an unexplored design space for creating technologies that can assist health practitioners who treat patients with depression. Particularly, 2 design opportunities are available: (1) designing technologies to help health practitioners collect accurate and real-time data about patients’ depression status and (2) designing technologies to help health practitioners interpret and incorporate nontraditional depression symptoms in their current treatment methods.

#### Design Opportunity 1: Designing Technologies to Help Health Practitioners Collect Accurate and Consistent Data

Treating depression can be difficult because the severity of the condition is mostly based on the perceptions of the patient and the clinician. Currently, patients provide information through their interaction with the health practitioners during office visits and through information that is recorded in their journals. These journals are often incomplete and do not provide the health practitioner with information recorded on a daily basis. When the health practitioners were asked to generate an indicator list that would be helpful in guiding the treatment of depression, many of the health practitioners listed patients’ daily and social functioning activities (Table 2). This was also reflected throughout the interviews with the health practitioners.

A level of functioning [would be helpful in guiding the treatment of depression].

Mental health practitioner 3

[I would like to know] how they’re acting, whether it’s a normal thing for them, or whether it’s a new change, or a sudden change, or a gradual change....

Nurse 4

And during the day, several times of, kind of a mental pulse taking, how are they doing, how are they facing life, how are their emotions, basically, during that day.

Nurse 5

Health practitioners rely on patient self-report to accurately assess the patient’s condition and develop treatment plans. Consumer health technology has a great potential to help health practitioners collect accurate data on an ongoing basis that can be used to help treat depression.

#### Design Opportunity 2: Designing Technologies to Help Health Practitioners Use Nontraditional Depression Indicators

Typical depression indicators include depressed mood, loss of interest or pleasure, irregular sleeping and eating habits, and behavioral changes. However, when health practitioners were asked to generate a list of indicators that would be helpful in guiding depression treatment, many of the health practitioners suggested nontraditional indicators (Table 2) and strongly believed that overall patients’ changes in behavior would be less helpful in guiding depression treatment. The generated list and interviews revealed that medical practitioners were interested in using atypical indicators such as the perspective of the family and friends of the patients to help guide treatment of depression.

someone else’s objective observations of the patient’s symptoms [would help guide treatment].

Nurse 4

...perception of those with whom the patient lives or works [would help guide treatment].

General practitioner 9

The mental health practitioners also listed and pointed to an interest in using nontraditional indicators to help practitioners with a guide to the treatment of depression. Mental health practitioners were very interested in obtaining more information pertaining to patients’ medical and medication records.

[I would like to know more about] all the medications they are on. All the medications they have tried, whether they have worked or not.

Mental health practitioner 7

Certain symptoms or side effects of medication can be important, some medications can really raise heart rate or blood pressure and having those data if not daily, then at least 2 or 3 times a week, can be really important data.

General practitioner 9

Since health practitioners were interested in incorporating other indicators within their current treatment methods. Consumer health technology could help health practitioners collect and interpret nontraditional indicators that will provide a more accurate depiction of patients’ depression status.

### Barriers to the Use of Technology in Depression Treatment

#### Overview

Although health practitioners expressed an interest in technologies, they also expressed concerns about several potential barriers to using technology to help treat depression. These concerns should be taken into consideration when designing consumer health technologies to assist health practitioners in treating patients with depression.

For example, while health practitioners were in favor of incorporating technology in their practice, many were skeptical of how it could be implemented within their current work infrastructure:

I think patients would like [using technology], but I would really see that being problematic with [our company] because we just don't have the staff to be able to do that, number one, and we don’t have the hardware to be able to do that, number two. We’re kind of low tech here.

Nurse 4

#### Design Implication 1

Consumer health technology should enhance the treatment process but not make it more cumbersome for the patient or the health practitioner.

Some health practitioners were concerned that technology would interfere with the treatment process. However, many health practitioners tried to actively involve their patients in the treatment process by asking them to track specific information concerning their depression. This act of collecting data within the data that is already available provides insight into the patients’ motivation levels:

Part of the treatment is what they don’t remember, as much as what they do.

Mental health practitioner 1

Indeed, some health practitioners felt that technology would hinder this process and would not allow for active patient participation:

Does this take away from the actual patient doing something?

Nurse 6

#### Design Implication 2

Consumer health technology should not remove patients’ involvement in data collection and reflection opportunities that are important clinical indicators of patients’ depression status.

At the same time, health practitioners were concerned that patients’ adoption would be low because patients were prone to forgetting to use the tool provided. In particular, patients already have difficulty remembering things and thus do not complete the current treatment activities that are assigned by the clinicians:

A lot of their memory is so horrible they can’t remember when their first episode of depression or when their first hospitalization was.

Mental health practitioner 7

Memory impairment is a common feature of, especially certain types of, mood disorder. A lot of times people report they can’t store memory well or recall well.

General practitioner 9

#### Design Implication 3

To compensate for memory impairment, consumer health technology should initiate use and encourage patients to complete the proscribed treatment task.

In addition, there are some types of data that could provide health practitioners with a better understanding of their patient’s current mental health, to which health practitioners currently cannot always have access. For example, some health practitioners desired to obtain specific information about patients’ medication use and side effects.

[I would like to know more about] all the medications they are on. All the medications they have tried, and whether they have worked or not.

Mental health practitioner 7

[I would like to know] are they taking their medication?

General practitioner 8

While patients may report on their subjective perception of the impact of medication, technology could provide objective data about medication quality that allow the health practitioner to determine if side effects from various medications could be contributing to their patients’ depression.

While this type of data could automatically be collected by using technology, the second design implication is important and must be considered.

#### Design Implication 4

Consumer health technology could be used to automatically collect data that would provide a more complete picture of the patient’s status to health practitioners, yet there must be a balance between automatic data collection and manual tracking to keep patients actively involved in the treatment process.

Finally, while health practitioners felt that patients would be able to use the technology, they noted that its cost may be prohibitive with their patient population:

Some of them wish they had better access to technologies. Some of them know a lot more about their technology than I do...they don’t have the money for technology.

Mental health practitioner 7

#### Design Implication 5

Whenever possible, use the technologies that patients already own, or use low-cost, commonly available commercial technologies.

### Existing Diagnostic and Treatment Paradigms Suggested by Health Practitioners’ Practices

#### Previous and Current Treatment Indicators

According to the Diagnostic and Statistical Manual of Mental Disorders IV-TR (DSM IV-TR), the primary symptoms that are required for a depression diagnosis are (1) depressed mood and/or (2) markedly diminished interest in activities or loss of pleasure for an extended period of time (days/weeks). Other symptoms such as sleep quality, social interactions, and inability to focus are also typical indicators of depression. Because health practitioners historically used these indicators to diagnose and treat depression, it was not surprising that these indicators were seen as better indicators of depression when we arrived at our quantitative data. However, when we asked health practitioners to describe their current treatment practices in their own words during the interview, differences in practices across health practitioner types emerged. For example, medical practitioners (nurses and general practitioners) tended to report relying on patients’ physical symptoms, overall health, medication use, and financial health:

Well I’ll need to know their past medical history, any medications that they take, their social history including their home state, married versus single, with whom do they live, parents, and do they have children? [Also their] occupation, history of drug and alcohol use, smoking. We usually want to know [their] family history.

General practitioner 9

I always ask my patients about their social economic status, their finances, social support, how they’re doing with nutrition. I want to know what exactly they do to alleviate symptoms. I also want to know how they medicate themselves and treat themselves...if they’re doing any supplements and drug use...I also want to know about any outside sources of help that they may have, church organizations, outside support groups.

Nurse 6

In contrast, mental health practitioners tended to use a less medical-centric, more client-feeling-centered approach to treatment:

I practice a very client-centered approach to treatment, so I’m going start where the client is. Primary for me is how the person is feeling and doing.

Mental health practitioner 1

We just make sure we take some time to assess what the client feels their strengths are or their parent or whoever their collateral support is gathering their strengths and so those are identified from the very beginning and carried out through their treatment so that we can maximize on those if needed.

Mental health practitioner 2

Mental health practitioners described taking advantage of the time they have with patients (multiple 30-minute to 1-hour-long sessions) to get a deep insight into their unique situations and find treatment plans tailored to them, whereas medical practitioners’ approach focused on medical history and current medical status. These qualitative differences suggest the way in which each type of health practitioners deals with patients. While these differences are not surprising given health practitioners’ different goals, training, and resources (eg, medical practitioners have been trained in a medical model), it is worth noting that these differences were not apparent in the quantitative results, though they were apparent in the list of health practitioner-generated indicators (see Table 2). One of the strengths of the mixed-methods approach adopted in this study is the ability to more fully understand and explain quantitative results. In this case, no differences were observed in the ratings that health practitioners gave to a set of a priori identified indicators. However, health practitioners reported differences in using these indicators in practice.

#### Design Implication 6

Technology designs should take into account the type of health practitioner who will use it, so that the technology fits with current clinical needs and practices recognizing that these practices may differ by the type of health practitioner.

As an example of how this design implication would play out in practice, one could imagine creating different versions of technology support for different types of health practitioners. For example, the technology mode designed for medical practitioners could focus on enabling an understanding of patients’ emotional levels and family and friends’ perspective as well as patient information relating to nutrition and physical activities, while a technology mode designed for mental health practitioners should focus on enabling clients’ reflections related to their own strengths, or client-specific symptoms (eg, sexual interactions).

Notably, both types of technology described above focus on indicators not considered as typical indicators of depression in the existing treatment paradigm. Instead, health practitioners often discussed other indicators that they used during treatment that were broader than the indicators outlined in current treatment theories. Thus, current theories of depression treatment need to be extended, adapted, or supplemented to account for varying practices of health practitioners as well as additional indicators health practitioners use during treatment. Also, while health practitioners of all types understand the value of a range of indicators (standard and nonstandard), a gap can be felt when it comes to using those indicators in their practice.

#### Design Implication 7

Consumer health technology designs could help bridge the gap between theory and practice by assisting health practitioners in collecting, interpreting, and integrating nonstandard, patient-centered depression indicators, and understanding how these additional variables affect recovery.

## Discussion

### Principal Findings

The present study provided several insights from health practitioners that can inform the design of novel consumer health depression treatment technologies. In general, health practitioners expressed an interest in using technology to help treat depression but had concerns about their adoption by patients with depression. To successfully create consumer health treatment technologies for depression, these concerns must be addressed early on during the design phase. Health practitioners’ training backgrounds also differed and each health practitioner type relied on different indicators to treat depression. Flexible technologies that can accommodate these differences should be created. Finally, health practitioners reported using additional indicators that are not explicitly outlined in the DSM IV-TR to treat depression. To translate depression treatment theory into practice, technology should help health practitioners successfully integrate traditional and nontraditional indicators within their treatment methods.

This paper presented design opportunities and challenges in designing depression treatment technologies. However, the current study was limited to a small number of health practitioners. While we are confident in our findings related to the existence of differences across health practitioner types, interviews with additional health practitioners are needed to gain a more accurate perspective of the needs and practices of each health practitioner type. In addition, health practitioners’ perspectives represent only one aspect of the treatment whereas patients represent the other aspect. We plan to incorporate patients’ perspectives of technology through field studies and observations in the near future to evolve a set of guidelines into a framework that can aid in the design of consumer health depression treatment technologies.

### Conclusions

This study examined various treatment styles of two categories of health practitioners as they relate to treating depression. The study also helped gather perspectives of different health practitioners on using consumer health technology to help guide the treatment of depression. The health practitioners were found favorable to using technology to help guide depression treatment but had concerns about its actual implementation. Based on these concerns, several design implications that can be used to help health practitioners feel more comfortable with using technology to treat depression were identified. In addition, the differences within each health practitioner’s clinical practice and treatment methods were also observed. Each type of health practitioner has a unique treatment method and relies on different depression indicators to guide treatment of depression. Technology should reflect these differences and be flexible enough to accommodate each health practitioner’s training background and clinical practices. Because health practitioners reported using additional indicators that were not outlined in the DSM IV-TR, technology should also strive to help various types of health practitioners understand, interpret, and integrate depression indicators within their current treatment methods. The findings in this study highlight design opportunities and provide an understanding of how both patients and health practitioners can effectively and successfully influence the design of consumer health technologies for the treatment of depression.

## Abbreviations

DSM IV-TR: Diagnostic and Statistical Manual of Mental Disorders IV-TR
